# Turkish Validity and Reliability Study of the InovSafeCare Scale for the Prevention and Control of Healthcare‐Associated Infections among Undergraduate Nursing Students: A Methodological Study

**DOI:** 10.1002/nop2.70705

**Published:** 2026-08-06

**Authors:** Fatma Birgili, Nezihe Bulut Uğurlu, Amaia Yurrebaso, Güllü Yazkan

**Affiliations:** ^1^ Department of Nursing Muğla, Health Sciences Faculty Muğla Sıtkı Koçman University Muğla Turkey; ^2^ Departamento de Psicología Social y Antropología, Centro de Investigación en Derechos Humanos y Políticas Públicas (CIDH) Universidad de Salamanca Salamanca Spain; ^3^ Department of Nursing Muğla, Health Sciences Institute Muğla Sıtkı Koçman University Muğla Turkey

**Keywords:** healthcare‐associated infections, InovSafeCare Scale, nursing students, Turkey, validity and reliability

## Abstract

**Aim:**

Healthcare‐associated infections are a major concern worldwide because they prolong hospital stays, increase costs for healthcare systems and negatively impact patients' health. Therefore, the aim of this study is to determine the Turkish Validity and Reliability of the InovSafeCare Scale for the prevention and control of healthcare‐associated infections among undergraduate nursing students who provide patient care services.

**Design:**

A methodological study design was used in this study.

**Methods:**

Study was conducted with the participation of 1149 nursing students from the Muğla and Fethiye University Nursing Departments between March and May 2025. Data were collected via a face‐to‐face survey. Data were analysed using Lawshe content validity ratio, Kaiser‐Mayer‐Olkin coefficient, Bartlett test, exploratory factor analysis, principal component analysis, Varimax factor rotation method, confirmatory factor analysis, Cronbach's *α* internal consistency coefficient, convergent validity, discriminant validity, test–retest and Spearman correlation coefficient tests.

**Results:**

InovSafeCare Scale was determined to have a 13‐factor structure. Composite reliability values were above 0.80 for all factors. The average explained variance values, an indicator of convergent validity, were at an acceptable level (≥ 0.50) for most factors. The Kaiser‐Meyer‐Olkin coefficient of the scale was 0.944, indicating excellent suitability for factor analysis of the sample size. Bartlett's test of sphericity (*χ* ^2^ = 5.60 × 10^4^, df = 3321, *p* < 0.001) was suitable for factor analysis of the correlation matrix. Thirteen factors explained 62.4% of the variance. Cronbach's *α* values of all factors ranged from 0.798 to 0.942, and the Cronbach's *α* coefficient of the scale was 0.942. Confirmatory factor analysis findings confirmed the 13‐factor structure of the scale in a statistically significant and strong manner (*p* < 0.05). The 2‐week test–retest reliability of the scale (*n* = 110) was also satisfactory (ICC = 0.878).

**Conclusion:**

The InovSafeCare Scale is a valid and reliable measurement tool that can be used to prevent and control healthcare‐associated infections among undergraduate nursing students who provide patient care.

**Patient or Public Contribution:**

No patient or public contribution.

## 1 Introduction

Healthcare‐associated infections (HCAIs) are infections that are not present or incubating at the time of care delivery and can arise as a result of healthcare interventions (CDC 2002; ECDPC 2020; WHO 2004). HCAIs are preventable infections, but with an incidence of between 7% and 10%, they pose a significant threat to the quality of care worldwide (Zimmerman et al. 2020; Alcan and Dolgun 2019; Rupam 2020). HCAIs are a cause of considerable concern worldwide due to their prolonged hospital stay, increased healthcare system costs and adverse effects on patient health (ECDPC 2020; Gastaldi et al. 2024; WHO 2007). A prevalence study conducted by WHO in 55 hospitals in 14 countries found that 8.7% of inpatients acquired the infection through hospital‐acquired means (Cohen et al. 2010). HCAIs can result in functional impairment, emotional distress, decreased quality of life, and death in patients, prolonged hospital stays, and increased disease costs (Arli and Bakan 2017; WHO 2016, 2011; Karahan et al. 2019; Sharma et al. 2020). The rise of antimicrobial resistance (AMR) has further exacerbated the challenges associated with the prevention and management of HAIs, as many pathogens responsible for these infections have evolved resistance to conventional treatment options. HCAIs are hospital‐acquired infections and are of critical importance for patient safety (Parreira et al. 2022). Each year, approximately 4 million patients in Europe acquire healthcare‐associated infections, 37,000 of which result in death (ECDPC 2020). The ECDPC estimates that approximately 30% of healthcare‐acquired infections could be prevented through surveillance systems, prevention and hygiene control programmes (ECDPC 2020). This constitutes undisputed preventive content for various professional groups in healthcare settings and essential academic content for all healthcare students (Díaz and Cadena 2003; González 2020). Brosio et al. (2017) found in a study that nursing students lacked knowledge about HCAI (Brosio et al. 2017). Furthermore, nursing students actively participate in practices in healthcare settings and, during this time, are at high risk of infection transmission due to direct contact with potentially infectious patients and their work with blood and body fluids (Díaz and Cadena 2003; González 2020). Despite preparations for HCAI in laboratory and simulated practice training, perceptions and attitudes regarding infection risks, and the use of personal protective equipment (PPE) remain inadequate (Parreira et al. 2022; Siesto 2017). Therefore, nursing students are exposed to highly contaminated environments through contact with patients/patient relatives or through physical contact, and it is estimated that more than 40% are infected (Kızıltepe and Yılmaz 2022).

Within the scope of Health Services and Infectious Diseases (IPC), education should be accompanied by guidelines on the essential components of infection prevention and control (IPC), based on priority topics identified by the World Health Organization (2020), and should include assessment strategies and tools. Approximately 50% of nursing education should be developed within a clinical context, focusing on learning within a team and, in direct contact with students and the public, on how to organize, provide, and evaluate the necessary care using the knowledge, capacities and skills acquired by the student (Rodríguez‐García 2019). The content to be taught in the practice sessions is theoretical and practical and should be developed by both academic and clinical instructors, and the content and learning process should be adapted to each context (Güler et al. 2024). Clinical instructors or supervisors play a crucial role in the teaching and learning process by creating a competent learning environment where other professionals in the care setting also participate, without sharing their educational responsibilities, interactions, habits, traditions and working conditions (Rodríguez‐García 2019).

The introduction of clinical practice aims to teach students the practical skills necessary to provide competent care, as the connection between theory and practice facilitates the assimilation of knowledge and the development of professional skills. Practices must still adhere to safety standards, meaning they must be based on interventions aimed at preventing or minimizing harm from patient care (Joint Commission on Accreditation of Healthcare Organization 2015). By clinical practice, we mean the caring function of the nursing profession, which encompasses all functions and activities carried out to address health and illness needs. Safe healthcare delivery practices must be based on adequate epidemiological surveillance systems that include multifaceted actions by multidisciplinary teams that support safe professional practices (Dener and Elçin 2022).

In addition to the educational organization itself (content, planning, guidance and process control), learning strategies can be influenced by each student's unique situation, both at the individual level (student's cognitive, motivational and behavioural levels) and the environment or learning context (patients, peers, other professionals, teachers, health centre structure and practices) (Tuğrul 2025). Indeed, a study with student nurses showed that the best predictor of hand hygiene compliance was their mentors' adherence to it (Şahbaz and Adana 2022). A study conducted in two paediatric hospitals in the United States during the pandemic revealed that 100% hand hygiene compliance (*n* = 72 healthcare workers) was achieved, a result interpreted as encouraging (Wong et al. 2020). Another study reported that before the pandemic, monthly EH compliance across all hospital units was 54.5% in September, while during the pandemic, compliance reached 92.8% and 100% in cohort units (Makhni et al. 2021). A study conducted in Turkey found this rate to be 100%, with 62.9% reported using hand hygiene (Birgili et al. 2023). Students consciously/unconsciously analyse the characteristics of the task, the resources available for its execution, and the limitations inherent in both the personal and learning context (Yesilyurt 2020). In short, preventive practice does not depend solely on knowledge or availability of preventive resources. Students must be motivated to implement them (Birgili et al. 2019; Birgili and Uğurlu 2019). This performance is a performance that, in addition to cognitive variables, can also be influenced by other variables related to the student's behavioural style, personality traits, the type of love and emotion he or she experiences at that time, and the motivations that push him or her to perform the task.

Taking all of these broad factors into account, as well as the ecosystem in which students develop their competencies, can be the basis for proposing an HCAI prevention and control ecological system and can reveal what the environment requires for a nursing student. An ecosystem can be thought of as an interactive system between organisms and their environment. This ecosystem also encompasses social practices. The human ecosystem is an interactive environment within the context of individuals, groups and cultures (Baycan 2024). This ecosystem has been used in many different fields, including business, education, media and healthcare with the Workforce Ecosystem Model. Specifically, this system consists of personnel, workflow design, personal/social factors, the physical environment and organizational factors (Chang et al. 2023). Based on this information, it is proposed that the student ecosystem for HCAI control and prevention practices should include three key factors that work as a collaborative bond for participation in safe care learning and performance. These are: educational and work environment, culture and beliefs; and being a student, practitioner and agent of change (Yurrebaso Macho et al. 2021). In this context, what factors constitute the student healthcare‐related disease control and prevention ecosystem?

A European Consortium developed the InovSafeCare Questionnaire to examine nursing students' perceptions, attitudes, and perceived performance regarding their teaching and learning experiences in health and care‐associated infection (HCAI) prevention and control. This scale provides an opportunity to examine and understand the current status of healthcare‐associated infection prevention and control from the perspective of the educational environment, healthcare settings, and nursing students' attitudes, beliefs and performance (Yurrebaso Macho et al. 2021).

Given that educational environments, professional socialization processes, and infection prevention cultures may differ across healthcare and sociocultural contexts, the cross‐cultural adaptation of psychometric instruments requires not only linguistic equivalence but also conceptual and structural evaluation within the target population. Accordingly, a European Consortium developed the InovSafeCare Questionnaire to examine nursing students' perceptions, attitudes, and perceived performance regarding their teaching and learning experiences in health and care‐associated infection (HCAI) prevention and control. This scale provides an opportunity to examine and understand the current status of healthcare‐associated infection prevention and control from the perspective of the educational environment, healthcare settings, and nursing students' attitudes, beliefs and performance.

Given that educational environments, professional socialisation processes, and infection prevention cultures may differ across healthcare and sociocultural contexts, the cross‐cultural adaptation of psychometric instruments requires not only linguistic equivalence but also conceptual and structural evaluation within the target population. Accordingly, slight structural variations in the factorial configuration of psychometric instruments may emerge across cultural contexts without necessarily compromising the conceptual integrity of the construct being assessed.

Therefore, this study aimed to translate the InovSafeCare Questionnaire into Turkish for use among undergraduate nursing students and to establish its validity and reliability in Turkish. The InovSafeCare Questionnaire will contribute to establishing a theoretical framework that can guide future improvement practices for safe practice among undergraduate nursing students and slight structural variations in the factorial configuration of psychometric instruments may emerge across cultural contexts without necessarily compromising the conceptual integrity of the construct being assessed.

Therefore, this study aimed to translate the InovSafeCare Questionnaire into Turkish for use among undergraduate nursing students and to establish its validity and reliability in Turkish. The InovSafeCare Questionnaire will contribute to establishing a theoretical framework that can guide future improvement practices for safe practice among undergraduate nursing students.

## 2 Methods

### 2.1 Study Design and Procedure

The study is a methodological study. This study tested the validity and reliability of the Turkish version of the InovSafeCare Questionnaire, which was originally in English. The STROBE checklist was used to report the research context, study design, methods and results of the study.

### 2.2 Conceptual Framework and Item Generation

In the study, the ‘Nursing Students' Perceptions on Healthcare‐Associated Infection Control and Prevention Teaching and Learning Experience (InovSafeCare)’ scale, which was created by the researchers in line with the literature, and the questionnaire form containing the demographic information of the nursing students, were used as the data collection tool (Yurrebaso Macho et al. 2021).

#### 2.2.1 Nursing Students' Perceptions on Healthcare‐Associated Infection Control and Prevention Teaching and Learning Experience (InovSafeCare) Scale

The InovSafeCare Scale, originally in English, was validated and reliable by Yurrebaso Macho et al. (2021). The scale is a 93‐item, 5‐point Likert‐type scale with 14 factors. Factors: I: Are the following Healthcare‐Associated Infection (HAI) topics addressed in your educational institution?, II: How is the content on HAI prevention and control presented in your educational institution?, III: How are practical contents related to HAI prevention and control taught at your educational institution?, IV: What are the HAI prevention and control measures in the Healthcare Institutions where you performed your clinical placements?, V: What is the HAI prevention and control culture in the Healthcare Institution where you were assigned to the clinic?, VI: What are your attitudes towards infection risks in your Healthcare Institutions?, VII: During your clinical placements, did you have any objections to healthcare‐acquired infection prevention and control measures?, VIII: How confident are you in your ability to follow safety measures for HAI prevention and control?, IX: How motivated do you feel about your future as a nurse?, X: To what extent have the following affected you in the last 4 months?, XI: During your clinical placements, do you get distracted and/or lose focus on your tasks due to the following reasons?, XII: What prompted you to follow HAI prevention and control procedures?, XIII: Did any of the following situations occur during your clinical placements?, XIV: Where did you learn about HAI prevention and control? Likert type: Items I–IX, XII–XIV: ‘1 – strongly disagree, 2 – partially disagree, 3 – neither agree nor disagree, 4 – partially agree, 5 – strongly agree’. Items X, XI: ‘1 – never, 2 – rarely, 3 – sometimes, 4 – often, 5 – always’.

It is a self‐report questionnaire that was back‐translated from English to Turkish and validated by 18 experts in the field. Its adaptation and construct validity were conducted using exploratory factor analysis (EFA), which allowed the identification of item clusters and the underlying dimensions of the construct under study, and the assessment of item validity using statistical methods in the Turkish context. In this study, 1149 questionnaires randomly collected from nursing students in two Health Sciences Faculties at Muğla Sıtkı Koçman University were analysed, following the methodological recommendations of Yurrebaso Macho et al. (2021).

### 2.3 Language Validity

The InovSafeCare scale has been translated to Turkish by back‐and‐forth translation for language validity in accordance with the WHO's ‘Translation and Adaptation of Tools’ Guidelines.

The original English‐Turkish translation of InovSafeCare was completed by five independent translators (five experts from the nursing field fluent in both languages). The five initial translated versions were reviewed by researchers in collaboration with consultants, and after reaching a consensus, the most appropriate expressions were combined into a single draft. Subsequently, translations from Turkish to English were performed (by five separate experts from the nursing field fluent in both languages), and the final translations were combined by researchers to create a single tool. The Turkish language and literature were checked by a linguistics teacher for clarity of expression and spelling.

### 2.4 Content Validity

The Lawshe technique was used to assess the content validity of the study. This is a technique in which experts evaluate the relevance of each item of a measurement tool to the relevant topic or domain (Romero Jeldres et al. 2023). Two rounds of content validity assessment were conducted to determine whether each preliminary item represented the measurement competence of both nurse academics and nursing students regarding the prevention and control of healthcare‐associated infections. A total of 18 experts, including 10 nurse academics, five nurse practitioners, and three final‐year nursing students, were consulted to provide their opinions on the content validity of the items. Eighteen experts were asked to rate the language translation accuracy of 93 translated items on a 1 to 4‐point scale using the InovSafeCare Scale, where 1: not appropriate, 2: somewhat appropriate (item/phrase revision needed), 3: quite appropriate (appropriate but minor changes needed), and 4: extremely appropriate. The scores obtained from the experts were as follows: 15/18 experts (83%) received 3–4 points, and 3/18 (17%) received 2 points. The Content Validity Index (CVI) was found by dividing the number of experts who received 3–4 points from the items by the total number of evaluations. Necessary corrections were made to the survey using the expert comments. Then, the content validity ratio CVI for each item and the CVI for the overall scale were calculated (the CVI of the items ranged from 0.857 to 1.000, and the CVI of the survey was 0.951) (Lawshe 1975). According to the Lawshe technique, the CVI of the scale items for 18 experts should be at least 0.42 at the *α* = 0.05 significance level (Ayre and Scally 2014). To determine the comprehensibility of the Turkish scale items, which were created after translation and content validity testing, a pilot study was conducted with 10 nursing students who met the acceptance criteria. Since no feedback was received regarding the scale items after the pilot study, the form created was used in the research. The data obtained from the pilot study were not used in statistical calculations.

### 2.5 Participants

To collect data, a face‐to‐face survey was administered to undergraduate nursing students studying in the nursing departments of two different health sciences faculties at a university in a province in Türkiye. The study participants were nursing students from these two different nursing departments, and a stratified sampling method was specifically used for this population (1460 students). Since it is recommended to reach a group size of at least 5–10 times the number of items when adapting a scale to another culture (Pallant 2020), the ‘InovSafeCare’ Scale contains 93 items, and therefore, 1149 nursing students constituted the study sample. To increase the generalizability of the study findings, a Systematic Random Sampling Method was also used for stratification. Efforts were made to achieve similar proportions of nursing students from each class. Inclusion criteria for participants were as follows: (1) being a student at the schools where the study was conducted during the study period, (2) having studied for at least one semester, (3) having performed hospital practice in the last 2 months, and (4) participating voluntarily. Additionally, incompletely filled questionnaires were not included in the study.

### 2.6 Data Collection

Data for the study were collected between March 2025 and May 2025 in the nursing departments of two different health sciences faculties of Muğla Sıtkı Koçman University. A 93‐item questionnaire including participant characteristics (3 questions) and the items of the InovSafeCare Scale was used for data collection. Data were collected via face‐to‐face interviews by four researchers responsible for data collection. The researchers provided information about the study to the participating students during their free class hours, and participants who agreed to participate were asked to sign a written consent form. The data collection form was explained in detail to the participants, and they were asked to fill out the data collection forms, which took 30–35 min. The completed forms were collected from the students by the researchers in sealed envelopes. Test–retest analysis was conducted for the reliability analysis of the draft scale (Streiner et al. 2024). For this purpose, 110 randomly selected nursing students were asked to fill out the scale again after 2 weeks.

### 2.7 Data Analysis

SPSS 30.0 and AMOS 24.0 package programmes were used to analyse the data obtained in the study. First, descriptive statistics (mean and standard deviation) as well as skewness and kurtosis coefficients were calculated for all scales used in the study. According to Kalaycı (2005), skewness and kurtosis values within ±1.96 indicate that the data are normally distributed. Considering this criterion, it was determined that the research data did not exhibit any significant deviation from a normal distribution.

Cronbach's Alpha coefficients and item‐total correlations were examined in the reliability analysis of the scales, and the internal consistency levels of all scales were found to be acceptable and high.

To assess construct validity, exploratory factor analysis (EFA) was initially conducted using a data‐driven approach in order to identify the latent structure emerging from the Turkish sample. Subsequently, the resulting factor solution was evaluated considering not only statistical criteria, but also conceptual coherence, interpretability, and theoretical consistency with the dimensions represented in the original InovSafeCare model. Finally, confirmatory factor analysis (CFA) was performed to assess the adequacy and stability of the resulting factorial structure.

Principal Axis Factoring (PAF) and Maximum Likelihood methods are frequently recommended for estimating latent structures in psychometric studies. However, principal component analysis (PCA) is also a widely used method, particularly in the exploratory phase of scale adaptation studies, to examine the factor structure of translated items and obtain an interpretable factor solution. In this study, a Turkish adaptation of a multidimensional scale consisting of approximately 82 items was carried out, and the analyses were conducted on a large sample of 1149 individuals. It is reported in the literature that PCA and common factor analyses (e.g., PAF) can produce similar factor structures and factor loadings in large samples and multi‐item scales. Therefore, PCA was preferred to reveal the factor structure of the Turkish form of the scale in the exploratory phase. However, the obtained factor structure was not based solely on PCA results. After the exploratory analysis, the proposed factor structure was tested with Confirmatory Factor Analysis (CFA), and it was shown that the 13‐factor model had acceptable fit indices. Therefore, PCA was used only in the exploratory phase, and the structural validity of the scale was supported by the DFA results. In cases where a high level of correlation is expected between factors, oblique rotation methods such as Direct Oblimin or Promax may be preferred. However, although the factors obtained in our study represent sub‐dimensions of the same theoretical structure, the correlations between factors were generally found to be low to moderate. The highest correlation coefficient between factors was *r* = 0.696, and lower levels of relationships were obtained between many sub‐dimensions. In addition, it was observed that the correlation coefficients were quite low in some factor pairs (e.g., *r* = 0.012, *r* = 0.008 and *r* = 0.038). These findings indicate that the factors are distinguishable structures. Therefore, Varimax, a simpler, more interpretable, and widely used orthogonal rotation method in the literature, was preferred.

The criteria used for item selection were that factor loadings should be ≥ 0.30, the difference between factor loadings in overlapping items should be at least 0.10, and factors with low explained variance and limited theoretical relevance should be carefully evaluated (Büyüköztürk 2020; Karagöz 2021). The item removal process was not based exclusively on statistical criteria. In addition to factor loadings and cross‐loading patterns, the conceptual interpretability and theoretical coherence of the emerging dimensions were also considered during the adaptation process. This approach aimed to preserve the conceptual integrity of the original construct while ensuring an adequate representation of the latent structure within the Turkish educational and sociocultural context.

Goodness of fit indices (*χ* ^2^/df, RMSEA, CFI, SRMR) were evaluated within the scope of CFA, and the scales were found to have a good level of fit. In addition, CR and AVE values were examined for convergent validity, and MSV and ASV values for discriminant validity.

Pearson correlation analysis was conducted to determine the relationships between variables, and the level of relationships was interpreted according to Büyüköztürk (2020) (0.00–0.30 as low, 0.30–0.70 as medium and 0.70–1.00 as high).

The consistency of the InovSafeCare Student Perception Scale, administered at two different times, over time was calculated using Intraclass Correlation Coefficient (ICC) analysis. Significance levels of 0.05 and 0.01 were used in all analyses in the study.

### 2.8 Ethical Considerations

Permission was obtained from Muğla Sıtkı Koçman University Medical and Health Sciences Ethics Committee to conduct the research (Date: 30.09.2024, Protocol No: 240072, Decision No: 105 Number: 72867572‐050.01.04‐200). From the Faculty of Health Sciences of Muğla Sıtkı Koçman University (Date: 16.04.2024, Number: E 10323300‐604 01‐744030). In addition, permission was received from Amaia Yurrebaso Macho via email for the scale used in the validity and reliability study. All participants were informed about the purpose of the study before data collection, and they were assured that the data would be kept confidential and used only for scientific purposes. Additionally, participants were asked to sign a written, informed consent form. Throughout the research process, the principles of the Helsinki Declaration, research and publication ethics were carefully observed.

## 3 Results

### 3.1 Characteristics of Participants

A total of 1149 undergraduate nursing students participated in this study. When the socio‐demographic characteristics of the students were evaluated, it was seen that 73.9% of the participants were students in the Nursing Department of Muğla, and 26.1% were students in the Nursing Department of Fethiye. The majority of the students were between 18 and 23 years old (91%). 65.4% of the participants were female and 34.6% were male. When the distribution of grade levels was examined, 25.2% of the students were in their first year, 27.8% in their second year, 26.9% in their third year, and 20.1% in their fourth year.

The total mean score of the scale was 3.46 ± 0.48, indicating that the students' perceptions of the teaching and prevention of healthcare‐associated infections were above the moderate level. When the subscales were examined, the HE‐Theory subscale, which reflects the students' theoretical knowledge level, had one of the highest mean values at 3.99 ± 0.81. Similarly, the high means in the P‐Attitude (4.07 ± 0.75) and IMotive‐EMotive (4.00 ± 0.79) subscales indicate that students have positive attitudes towards infection risk and strong motivation to comply. Conversely, the low means in the SR‐Practice (2.39 ± 1.06), which measures clinical protocol violations, and the P‐Objection (2.70 ± 1.05), which assesses attitudes towards inappropriate practices, indicate that students are less inclined to engage in behaviours that pose a risk to infection control. The lower means in the HE‐Practice (3.02 ± 1.08), which represents practical teaching, and the Distract (3.18 ± 0.85), which assesses distracting clinical elements, suggest that students may need support in their clinical learning processes.

When the skewness and kurtosis values for the scale and subdimensions are examined, it is seen that all values fall within the ±1.96 range recommended by Kalaycı (2005) for a normal distribution. The fact that the total score of the InovSafeCare Student Perception Scale and the skewness and kurtosis coefficients for all subfactors are within these limits indicates that the variables exhibit a normal distribution.

### 3.2 Reliability Analysis

The EFA conducted on the InovSafeCare Student Perception Scale determined that some items should be removed from the scale due to cross‐loading or insufficient factor variance.

Initial exploratory analyses yielded alternative factorial solutions with a higher number of factors. However, several of these emerging factors consisted of a very limited number of items and contributed minimally to the overall explained variance. Therefore, the factorial solutions were evaluated considering both psychometric robustness and conceptual interpretability, with the aim of identifying the most theoretically coherent and structurally stable representation of the construct within the Turkish context.

In subsequent loading analyses, items m51, m70, m71, m50, m69 and m74 were found to have cross‐loadings and were removed from the scale. Additionally, one emerging factor composed of only two items showed limited contribution to the overall explained variance and insufficient structural stability. Considering both psychometric recommendations regarding factor robustness and the conceptual coherence of the construct, this factor was not retained in the final factorial solution.

As a result of these arrangements, the scale was purified from overlapping and low‐loading items and reached a final 13‐factor structure that is theoretically compatible, reliable and best explains the total variance.

The scale's Kaiser‐Meyer‐Olkin (KMO) sampling adequacy coefficient was found to be 0.944, indicating that the sample size was perfectly suitable for factor analysis. The Bartlett test of sphericity (*χ* ^2^ = 5.60 × 10^4^, df = 3321, *p* < 0.001) determined that the correlation matrix was suitable for factor analysis. These findings demonstrate that the data meet the statistical conditions necessary to reveal a multifactorial structure.

Examination of the eigenvalues after rotation revealed that the scale exhibited a 13‐factor structure, and these factors explained a total of 62.4% of the variance. It was determined that the variance explained by individual factors ranged from 2.9% to 10.5%, with each dimension contributing significantly to the overall scale (Table 1). These findings support the multidimensional structure of the scale and indicate that the resulting factorial solution provides a statistically adequate and conceptually coherent representation of nursing students' perceptions regarding HCAI prevention and control within the Turkish educational and clinical context.

**TABLE 1:** KMO, Bartlett Test, eigenvalue (after rotation) and variance results of InovSafeCare Student Perception Scale.

Scale English	Scale and its factors	KMO	Bartlett's test			After rotation	
			*χ* ^2^	df	*p*	Core values	Variance (%)
InovSafeCare Scale	InovSafeCare Scale	0.944	5.60 × 10^4^	3321	*0.000		62.4
HE_Theory1	1. Chain of Infection					8.60	10.5
HE‐Approach	2. HCAI Teaching Approach					2.34	2.9
HE‐Practice	3. HCAI Application Instruction					4.23	5.2
HI‐Practice	4. Clinical HCAI Applications					3.63	4.4
HI‐Culture	5. Clinical HCAI Culture					2.97	3.6
P‐Attitude	6. Infection Risk Attitudes					4.12	5.0
P‐Objection	7. Attitudes Towards Inappropriate Practices					3.03	3.7
C‐Motive	8. Professional Motivation					3.25	4.0
F‐Fatigue	9. Fatigue					4.05	4.9
D‐Distract	10. Distracting Factors					3.89	4.7
IMotive + EMotive	11. HCAI Compliance Motivations					2.77	3.4
SR‐Pract	12. Protocol Violation Behaviours					4.84	5.9
K‐Source	13. HCAI Information Sources					3.46	4.2

Abbreviation: KMO, Kaiser‐Meyer‐Olkin.

*p < 0.01.

The EFA for the InovSafeCare Student Perception Scale determined that the scale has a 13‐factor structure. Cronbach's *α* values for all factors ranged from 0.798 to 0.942, and the Cronbach's *α* coefficient for the entire scale was 0.942 (Table 2). These values indicate that the scale has high internal consistency.

**TABLE 2:** Results of item factor loadings, reliability and item‐total correlation analysis of the InovSafeCare Student Perception Scale.

No	English	Items belonging to the scale	Factor loadings	Item‐total correlation	Cronbach's *α*
	*HE‐Theory*	*HCAI Theoretical Content*			*0.939*
1	HE_Theory1	1. Chain of infection	0.649	0.702	
2	HE_Theory2	2. Individual assessment of infection risk during patient admission and hospitalization/isolation	0.704	0.732	
3	HE_Theory3	3. Hand hygiene	0.747	0.745	
4	HE_Theory4	4. Respiratory tract infection	0.758	0.761	
5	HE_Theory5	5. Use of personal protective equipment (gloves, masks, gowns, etc.)	0.783	0.757	
6	HE_Theory6	6. Disinfection and sterilization (decontamination) of medical/clinical devices and equipment	0.745	0.709	
7	HE_Theory7	7. Proper cleaning (decontamination) of surfaces within the hospital	0.738	0.709	
8	HE_Theory8	8. Safe use of protective clothing	0.777	0.757	
9	HE_Theory9	9. Proper management of clinical waste	0.703	0.719	
10	HE_Theory10	10. Safe preparation and administration of intravenous medications	0.717	0.729	
11	HE_Theory11	11. Taking necessary precautions to protect against disease‐causing microorganisms in the hospital	0.724	0.728	
12	HE_Theory12	12. Additional precautions for specific transmission routes	0.651	0.661	
	*HE‐Approach*	*HCAI Teaching Approach*			*0.835*
13	HE_Approach1	13. There are compulsory courses aimed at developing competencies	0.645	0.635	
14	HE_Approach2	14. HCAI prevention and control topics are included in theoretical courses	0.666	0.652	
15	HE_Approach3	15. Instructors provide laboratory work or lectures to students	0.556	0.619	
16	HE_Approach4	16. Visual resources and other technologies are used to explain HCAI topics in classes	0.592	0.679	
17	HE_Approach5	17. Instructors discuss relevant HCAI prevention and control measures within the context of the course when appropriate	0.548	0.626	
	*HE‐Practice*	*HCAI Application Instruction*			*0.89*
18	HE_Practice1	18. Students have sufficient technological resources (e.g., mannequins, medical/clinical devices) to develop their competencies	0.701	0.684	
19	HE_Practice2	19. Laboratory courses have an adequate student‐to‐teacher ratio	0.802	0.759	
20	HE_Practice3	20. There are sufficient laboratory courses where I can develop my competencies	0.832	0.797	
21	HE_Practice4	21. Simulation is used as a learning method for students to develop their competencies in a structured environment	0.765	0.748	
22	HE_Practice5	22. I receive feedback from educators on practices related to HCAI prevention and control	0.669	0.673	
	*HI‐Practice*	*Clinical HCAI Applications*			*0.842*
23	HI_Practice1	23. Hand hygiene supplies are available in every patient and treatment room	0.499	0.540	
24	HI_Practice2	24. There is a ‘hand hygiene practice’ policy (e.g., no wristwatches, rings, or bracelets)	0.553	0.610	
25	HI_Practice3	25. Infection control nurses are also included in my clinical rotations	0.555	0.571	
26	HI_Practice4	26. Resources related to Healthcare AI are available to all healthcare professionals (e.g., equipment, protocols and regulations)	0.548	0.572	
27	HI_Practice5	27. Visual reminders are available in designated areas (e.g., corridors, treatment rooms) to increase hand hygiene compliance	0.651	0.631	
28	HI_Practice6	28. Alcohol‐based hand sanitizers are available in key areas (e.g., patient beds, clinic entrances)	0.673	0.649	
29	HI_Practice7	29. Personal protective equipment is available in key areas (e.g., in front of isolation room doors)	0.619	0.605	
	*HI‐Culture*	*Clinical HCAI Culture*			*0.873*
30	HI_Culture2	31. Healthcare workers follow safety protocols and procedures for HCAI prevention and control in the healthcare sector	0.488	0.642	
31	HI_Culture3	32. All staff members follow safety protocols and procedures for HCAI prevention and control	0.588	0.684	
32	HI_Culture4	33. I receive guidance on HCAI prevention and control from educators and clinical nurses	0.620	0.680	
33	HI_Culture5	34. Educators and clinical nurses encourage us to use available personal protective equipment	0.497	0.637	
34	HI_Culture6	35. If I do not comply with HCAI infection prevention and control measures, I receive feedback from educators and clinical nurses to improve my performance	0.661	0.715	
35	HI_Culture7	36. Nurses motivate me to comply with HCAI prevention and control measures	0.672	0.693	
	*P‐Attitude*	*Infection Risk Attitudes*			*0.863*
36	P_Attitude1	37. There is always a potential risk of HCAI in patient care	0.658	0.673	
37	P_Attitude2	38. As a student, I feel responsible for preventing and controlling the spread of infections	0.716	0.729	
38	P_Attitude3	39. I would refuse to care for patients if I could not comply with infection prevention and control measures in the healthcare sector	0.622	0.558	
39	P_Attitude4	40. I believe that all protocols for HCAI prevention and control should be followed	0.709	0.754	
40	P_Attitude5	41. I believe that my safety precautions contribute to preventing the spread of infections	0.708	0.735	
41	P_Attitude6	42. I value scientific advice more than my own opinion	0.564	0.549	
42	SelfEff1	48. I find it easy to follow safety protocols	0.410	0.472	
	*P‐Objection*	*Attitudes to Inappropriate Practices*			*0.838*
43	P_Objection1	43. I find some HCAI safety protocols unnecessary	0.709	0.652	
44	P_Objection2	44. I can express my reservations about the implementation of HCAI prevention and control measures, which I consider unnecessary	0.574	0.433	
45	P_Objection3	45. I find the use of so many disposable items unnecessary	0.746	0.737	
46	P_Objection4	46. I find personal protective equipment uncomfortable	0.754	0.741	
47	P_Objection5	47. I am concerned that educators and clinical nurses constantly monitor my HCAI infection prevention and control measures in the healthcare setting where I practice	0.720	0.0.646	
	*C‐Motive*	*Professional Motivation*			*0.912*
48	C_Motive1	52. I am happy with my career choice	0.868	0.815	
49	C_Motive2	53. My clinical rotations motivate me to work as a nurse	0.807	0.768	
50	C_Motive3	54. I am very eager to pursue my career as a nurse	0.887	0.855	
51	C_Motive4	55. Being a nurse will meet my expectations for the career I want to pursue	0.813	0.765	
	*Fatigue*	*Fatigue*			*0.893*
52	Fatigue1	56. I experienced academic overload	0.623	0.572	
53	Fatigue2	57. I experienced difficult times in my personal (private) life	0.772	0.704	
54	Fatigue3	58. My clinical rotations coincided with periods when I felt tired	0.765	0.711	
55	Fatigue4	59. I felt emotionally tired	0.863	0.805	
56	Fatigue5	60. I felt mentally tired	0.857	0.789	
57	Fatigue6	61. I felt physically tired	0.778	0.712	
	*Distract*	*Distractions*			*0.860*
58	Distract1	62. Cluttered rooms or wards	0.666	0.588	
59	Distract2	63. Too many patients in the same room/area	0.756	0.665	
60	Distract3	64. Too many demands from patients and/or family members	0.732	0.649	
61	Distract4	65. Communicating with patients in my own age group	0.592	0.546	
62	Distract5	66. Caring for patients with multiple conditions	0.739	0.664	
63	Distract6	67. Emotionally challenging cases	0.709	0.638	
64	Distract7	68. Having to perform more than one clinical task	0.710	0.646	
	*IMotive + EMotive*	*HCAI Compliance Motivations*			*0.862*
65	Imotive_EMotive8	76. I take more careful notes regarding my adherence to HCAI prevention and control procedures	0.687	0.649	
66	Imotive_EMotive9	77. Other team members will approve of my adherence to HCAI prevention and control procedures	0.798	0.773	
67	Imotive_EMotive10	78. I will ensure that other team members also follow the HCAI prevention and control procedures I have implemented	0.777	0.748	
68	Imotive_EMotive11	79. I will meet the HCAI prevention and control expectations of educators and clinical nurses	0.728	0.672	
	*SR‐Pract*	*Protocol Violations*			*0.898*
69	SR_Pract1	80. I forgot to follow required safety protocols	0.720	0.622	
70	SR_Pract2	81. I forgot to remove accessories I was wearing on my wrists (e.g., wristwatches, bracelets, rings)	0.741	0.689	
71	SR_Pract3	82. I did not wear gloves when necessary	0.818	0.783	
72	SR_Pract4	83. I did not wash and/or disinfect my hands when required	0.821	0.829	
73	SR_Pract5	84. I did not wear a face mask when required	0.817	0.769	
74	SR_Pract6	85. I cared for one patient after another without changing my gloves, mask, or other protective equipment	0.757	0.694	
75	K_Source1	86. I took a special course on HCAI prevention and control	0.510	0.539	
	*K‐Source*	*HCAI Information Sources*			*0.798*
76	K_Source2	87. From other professional courses (e.g., paediatric nursing, elder care nursing)	0.498	0.440	
77	K_Source3	88. From my classmates	0.560	0.531	
78	K_Source4	89. From simulation classes	0.609	0.552	
79	K_Source5	90. From clinical nurses during internships	0.709	0.598	
80	K_Source6	91. From educators during clinical practice	0.676	0.528	
81	K_Source7	92. From other healthcare professionals during clinical practice (e.g., physicians, pharmacists)	0.703	0.561	
82	K_Source8	93. From scientific studies and literature reviews (field‐related, procedures, articles)	0.623	0.509	
		InovSafeCare Student Perception Scale	0.942		

The HCAI Theoretical Content factor consists of 12 items, with factor loadings ranging from 0.649 to 0.783, and item‐total correlations ranging from 0.661 to 0.761. The factor's Cronbach's *α* value is 0.939, indicating a very high level of reliability. The HCAI Teaching Approach factor consists of 5 items, with factor loadings ranging from 0.548 to 0.666, and item‐total correlations ranging from 0.619 to 0.679. Cronbach's *α* coefficient of the factor was found to be 0.835. The HCAI Application Teaching factor consists of 5 items with factor loadings ranging from 0.669 to 0.832, and item‐total correlations ranging from 0.673 to 0.797. Cronbach's *α* value of 0.890 indicates a high level of reliability. The Clinical HCAI Applications factor consists of 7 items with factor loadings ranging from 0.499 to 0.673, and item‐total correlations ranging from 0.54 to 0.649. Cronbach's *α* value of this factor was found to be 0.842. The Clinical HCAI Culture factor consists of 6 items with factor loadings ranging from 0.488 to 0.672, and item‐total correlations ranging from 0.637 to 0.715. The Cronbach's *α* coefficient of the factor is 0.873, indicating a good level of internal consistency. The Infection Risk Attitudes factor consists of 7 items, factor loadings range from 0.41 to 0.716, and item‐total correlations range from 0.472 to 0.754. Cronbach's *α* value was determined as 0.863. The Attitudes Towards Inappropriate Practices factor consists of 5 items, factor loadings range from 0.574 to 0.754, and item‐total correlations range from 0.433 to 0.741. Cronbach's *α* value of the factor is 0.838. The Professional Motivation factor consists of 4 strong items; factor loadings range from 0.807 to 0.887, and item‐total correlations range from 0.765 to 0.855. The internal consistency coefficient of this factor is 0.912, which is quite high.

The Fatigue factor consists of 6 items, with factor loadings ranging from 0.623 to 0.863, and item‐total correlations ranging from 0.572 to 0.805. Cronbach's *α* coefficient for the factor was found to be 0.893. The Distracting Factors factor consists of 7 items, with factor loadings ranging from 0.592 to 0.756, and item‐total correlations ranging from 0.546 to 0.665. Cronbach's *α* was found to be 0.860. The HCAI Adaptive Motivations factor consists of 4 items, with factor loadings ranging from 0.687 to 0.798, and item‐total correlations ranging from 0.649 to 0.773. Cronbach's *α* coefficient was calculated as 0.862 (Table 2).

The Protocol Violation Behaviours factor consists of 6 items, with factor loadings ranging from 0.72 to 0.821, and item‐total correlations ranging from 0.622 to 0.829. Cronbach's *α* for this factor is 0.898. The HCAI Information Sources factor consists of 8 items. Factor loadings range from 0.498 to 0.709, and item‐total correlations range from 0.44 to 0.561 (Table 2). Cronbach's *α* coefficient is 0.798, which is at an acceptable level. Overall, these findings support the reliability and internal consistency of the multidimensional structure of the InovSafeCare Student Perception Scale within the Turkish context.

### 3.3 Confirmatory Factor Analysis (CFA)

Confirmatory factor analyses (CFA) were conducted to test the constructed measurement model and the validity of the EFA results. CFA findings supported the adequacy of the 13‐factor structure of the InovSafeCare Student Perception Scale, confirming the structural consistency of the factorial solution obtained through EFA. The 12 items under the first factor, ‘F1’, contributed to the model with high factor loadings (0.661–0.791), supporting the strong structure of the theoretical education dimension. The ‘F2’ factor was confirmed with 5 items, with loadings ranging from 0.630 to 0.785, demonstrating that teaching methods were significantly represented as a separate dimension in the scale. The ‘F3’ factor was confirmed with very high loadings ranging from 0.726 to 0.857, demonstrating that applied education is one of the strongest sub‐dimensions of the scale.

Seven items of the ‘F4’ factor related to clinical practices contributed significantly with loadings ranging from 0.618 to 0.703, confirming the multidimensional nature of infection control at the clinical level. The ‘F5’ factor showed loadings ranging from 0.700 to 0.763 with 6 items, supporting the notion that clinical safety culture is a robust sub‐dimension in the scale structure. The ‘F6’ factor measuring students' attitudes was confirmed with loadings ranging from 0.524 to 0.823 with 7 items. The ‘F7’ factor was supported with loadings ranging from 0.465 to 0.840 for 5 items, reflecting students' reactions to inappropriate infection control practices. Four items of the ‘F8’ factor were confirmed with very high loadings ranging from 0.815 to 0.911, demonstrating that the motivation dimension is one of the strongest factors of the scale. The ‘F9’ factor showed high loadings ranging from 0.573 to 0.890 with 6 items. Factor ‘F10’, measuring clinical distractions, loaded between 0.595 and 0.732 with 7 items. Factor ‘F11’, representing compliance motivation, was confirmed with loadings ranging from 0.721 to 0.850 across 4 items. Factor ‘F12’, based on student self‐reports, was supported with loadings ranging from 0.582 to 0.892 across 7 items, with item SR_Pract4 exhibiting the highest loading. Finally, factor ‘F13’, with 8 items, loaded between 0.493 and 0.691. Overall, the CFA findings support the structural adequacy of the multidimensional model and indicate consistency between the empirical factorial solution and the theoretical dimensions underlying the InovSafeCare construct.

To improve model fit, theoretically justifiable covariance modifications were applied between the error terms e58–e59, e13–e14 and e11–e12 during the analysis process. Following these modifications, confirmatory factor analysis results indicated that the model demonstrated an acceptable overall fit (*χ* ^2^ = 8935.493; SD = 3158; *p* < 0.001; CMIN/df = 2.829; RMSEA = 0.040; CFI = 0.893; SRMR = 0.055). The obtained goodness‐of‐fit indices indicate that the model is within the recommended limits in terms of both absolute and comparative fit.

### 3.4 Convergent and Discriminant Validity of the Scale

Table 3 presents the validity and reliability indicators obtained as a result of the confirmatory factor analysis of the InovSafeCare Student Perception Scale.

**TABLE 3:** InovSafeCare Student Perception Scale – CFA validity/reliability indicators.

Factor	CR	AVE	MSV	ASV
F1 – Theoretical Content	0.939	0.562	0.426	0.171
F2 – Teaching Approach	0.828	0.492	0.396	0.178
F3 – Practice‐Based Teaching	0.892	0.623	0.396	0.119
F4 – Clinical HAI Practices	0.844	0.437	0.640	0.212
F5 – Clinical Safety Culture	0.874	0.536	0.640	0.219
F6 – Infection Risk Attitudes	0.871	0.497	0.426	0.164
F7 – Objection to Unsafe Practice	0.842	0.524	0.341	0.061
F8 – Professional Motivation	0.914	0.726	0.160	0.075
F9 – Fatigue	0.895	0.591	0.216	0.030
F10 – Distraction Factors	0.857	0.463	0.216	0.041
F11 – Compliance Motivation	0.866	0.618	0.284	0.131
F12 – Protocol Violations	0.901	0.570	0.341	0.061
F13 – Knowledge Sources	0.803	0.370	0.224	0.100

Abbreviations: ASV, average shared squared variance; AVE, average variance extracted, CR, composite/construct reliability; MSV, maximum squared variance.

Composite reliability (CR) values were observed to be above 0.80 for all factors. These findings indicate satisfactory internal consistency across the multidimensional structure of the scale. The average variance extracted (AVE), an indicator of convergent validity, was at acceptable levels (≥ 0.50) for most factors. AVE values above the recommended threshold for factors F1, F3, F5, F8, F9, F11 and F12 support the convergent validity of these dimensions (Table 3). Although the AVE values for factors F4, F6, F10 and F13 were below the recommended threshold, the corresponding CR values remained acceptable, suggesting adequate construct reliability and partial support for convergent validity.

In terms of discriminant validity, the maximum shared variance (MSV) was observed to be lower than the AVE for most factors. These findings support the discriminant validity of the dimensions. Similarly, the fact that the average shared variance (ASV) values were lower than the AVE values also supports discriminant validity (Table 3).

Overall, these findings support the convergent and discriminant validity of the 13‐factor structure of the InovSafeCare Student Perception Scale and indicate that the instrument provides a psychometrically adequate and conceptually coherent assessment framework within the Turkish context.

### 3.5 Reliability Analysis

The intraclass correlation coefficient (ICC) values of the consistency of the InovSafeCare Student Perception Scale applied at two different times are presented in Table 4.

**TABLE 4:** Test–retest reliability (ICC) of InovSafeCare Student Perception Scale and its sub‐dimensions.

Scale and sub‐factors	ICC	95% confidence interval (lower–upper)	*p*
InovSafeCare‐Scale (Total)	0.878	0.818–0.918	< 0.001*
HE‐Theory	0.774	0.664–0.848	< 0.001*
HE‐Approach	0.736	0.607–0.822	< 0.001*
HE‐Practice	0.757	0.638–0.836	< 0.001*
HI‐Practice	0.786	0.682–0.856	< 0.001*
HI‐Culture	0.749	0.627–0.831	< 0.001*
P‐Attitude	0.717	0.580–0.810	< 0.001*
P‐Objection	0.607	0.415–0.735	< 0.001*
C‐Motive	0.911	0.868–0.940	< 0.001*
Fatigue	0.848	0.774–0.898	< 0.001*
Distract	0.900	0.851–0.933	< 0.001*
IMotive–EMotive	0.704	0.560–0.801	< 0.001*
SR‐Pract	0.826	0.741–0.883	< 0.001*
K‐Source	0.844	0.768–0.895	< 0.001*

*Note:* 0.50–0.75 = moderate, 0.75–0.90 = good, > 0.90 = excellent reliability is considered (Koo and Li 2016).

Abbreviation: ICC, intraclass correlation coefficient.

* *p* < 0.05.

The findings indicate that the overall score of the scale demonstrated good temporal stability (ICC = 0.878), supporting the consistency of the instrument over time. Among the subdimensions, C‐Motive (ICC = 0.911) and Distract (ICC = 0.900) showed excellent reliability, indicating highly stable responses across repeated measurements. In addition, HI‐Practice (0.786), HE‐Practice (0.757), Fatigue (0.848) and K‐Source (0.844) demonstrated good reliability levels. The P‐Attitude (0.717) and P‐Objection (0.607) dimensions showed moderate temporal consistency (Table 4).

Overall, the ICC findings support the test–retest reliability of the InovSafeCare Student Perception Scale and indicate that the instrument provides stable measurements across time within the Turkish context.

## 4 Discussion

Healthcare‐associated infections (HCAIs) continue to represent one of the most important challenges for healthcare systems worldwide due to their impact on patient safety, morbidity, mortality, healthcare costs and quality of care. Beyond their clinical implications, HCAIs also reflect organizational, educational and cultural deficiencies related to infection prevention and control practices. In this context, nursing students constitute a particularly relevant population because professional competencies, preventive behaviours and safety‐related attitudes begin to develop during undergraduate education and are reinforced through clinical socialization processes. Consequently, the availability of psychometrically robust instruments capable of assessing nursing students' perceptions, attitudes, motivations and behaviours regarding HCAI prevention and control is essential both for educational evaluation and for the development of safer healthcare cultures.

In the first phase of our study, national and international literature on infection prevention assessment scales developed for nurses and nursing students was reviewed. The aim was to evaluate the validity and reliability of the InovSafeCare Scale, developed by Amaia in Yurrebaso Macho et al. 2021, to assess the control and prevention of healthcare‐associated infections in the practice of Turkish nursing students. The findings of this study are consistent with previous studies validating similar instruments (Yurrebaso Macho et al. 2021; Ramos‐Meza et al. 2025; Turan et al. 2025; Çelik et al. 2026) and confirm that the InovSafeCare Scale is a robust tool for assessing infection control and prevention behaviours among nursing students in Türkiye. The results of this study showed that the Turkish version of the scale has strong linguistic and content validity with an overall CVI of 0.951. The increased reliability observed in the Turkish version may reflect careful linguistic and cultural adaptation, highlighted as a key factor in successful cross‐cultural scale validation (WHO 2014). Minor cultural adjustments were made to accommodate the context of Turkish nursing students. CFA supported the construct validity of the scale, with all item loadings exceeding the acceptable threshold of 0.30 and goodness‐of‐fit indices (CMIN/df = 2.829; RMSEA = 0.040; CFI = 0.893; SRMR = 0.055) indicating a good model fit (Sousa and Rojjanasrirat 2011; WHO 2014). In this study, Cronbach's alpha was calculated to be 0.942 and for all factors ranged from 0.798 to 0.942, indicating that the scale demonstrates high reliability. The large sample size (*n* = 1149), multidimensional scale structure, comprehensive psychometric analyses, and validity and reliability evidence are the strengths of our study. These findings demonstrate that the adapted scale is both a valid and reliable tool for assessing infection control and prevention competencies in Turkish nursing students.

The original InovSafeCare Scale was developed within a multinational European framework including nursing students from Portugal, Spain, Poland and Finland (Yurrebaso Macho et al. 2021). The original validation study demonstrated adequate psychometric properties and conceptual consistency across different healthcare and educational systems, supporting the multidimensional nature of the construct and its applicability within diverse European contexts. The scale was originally composed of 93 items distributed across 14 dimensions associated with educational environment, clinical culture, motivational processes, attitudes towards infection prevention and behavioural compliance with HCAI control measures. More recently, Samuel Ramos‐Meza et al. (2025), in a Peruvian sample of nursing students, also identified a reorganized factorial structure through exploratory factor analysis, resulting in a reduced factorial configuration with 11 dimensions. These findings collectively suggest that although the central conceptual domains underlying the InovSafeCare framework remain stable, the latent organization of these dimensions may vary according to sociocultural, educational and institutional contexts.

In the present study, the Turkish adaptation of the InovSafeCare Student Perception Scale resulted in a psychometrically adequate structure composed of 82 items distributed across 13 factors: Chain of Infection, HCAI Teaching Approach, HCAI Practice Instruction, Clinical HCAI Applications, Clinical HCAI Culture, Infection Risk Attitudes, Attitudes Towards Inappropriate Practices, Professional Motivation, Fatigue, Distracting Factors, HCAI Compliance Motivations, Protocol Violation Behaviours and HCAI Information Sources. Overall, the findings support the structural adequacy, reliability and conceptual coherence of the Turkish version of the instrument and reinforce its utility for evaluating nursing students' educational and clinical experiences regarding infection prevention and control.

Parallel analysis is one of the current methods recommended for determining the number of factors. However, in this study, the Kaiser eigenvalue (> 1) criterion was not the only criterion used to determine the number of factors. Eigenvalues, total variance explained, Scree Plot graph, factor loadings, cross‐loaded items, theoretical suitability of items, and the original factor structure of the scale were all considered together when deciding on the number of factors. Therefore, the number of factors was determined not based on a single statistical criterion, but by considering both statistical and theoretical evaluations. Furthermore, the factor structure obtained from the exploratory factor analysis (EFA) was subsequently validated with confirmatory factor analysis (CFA); when the model showed acceptable fit indices, high internal consistency coefficients (Cronbach's *α*), composite reliability (CR), average variance extracted (AVE) and discriminant validity indicators, the proposed 13‐factor structure was found to possess strong psychometric properties.

One of the most relevant findings of this study concerns the partial reorganization of the factorial structure during the adaptation process. Importantly, this reorganization should not be interpreted as a methodological weakness or as a loss of conceptual integrity of the original instrument. Cross‐cultural adaptation studies do not necessarily require an identical replication of the original factorial structure; rather, they seek to determine whether the central conceptual domains of the construct remain meaningful, interpretable and psychometrically stable within the target population. Educational systems, professional socialization processes, institutional hierarchies, and culturally mediated understandings of responsibility and compliance may substantially influence how preventive behaviours and infection‐control attitudes are cognitively organized by nursing students.

During the exploratory factor analysis process, several items demonstrated cross‐loadings or contributed minimally to the explained variance and were therefore removed from the final structure. Importantly, item elimination was not based exclusively on statistical criteria. In addition to factor loadings and variance contribution, the conceptual interpretability, theoretical consistency, and structural coherence of the emerging dimensions were systematically considered throughout the adaptation process. This methodological approach aimed to preserve the conceptual integrity of the original construct while ensuring that the resulting factorial solution adequately represented the Turkish educational and sociocultural context.

A particularly noteworthy finding was the integration of the original ‘Intrinsic Motivation’ and ‘Extrinsic Motivation’ dimensions into a single factor labelled ‘HCAI Compliance Motivations’. This result may reflect culturally mediated forms of professional motivation in which personal commitment, institutional expectations, collective responsibility, and externally reinforced professional norms are experienced as interconnected rather than clearly differentiated motivational systems. In highly structured educational and clinical environments, motivational processes related to infection prevention behaviours may emerge through a more integrated framework linking personal responsibility, professional identity, peer expectations and institutional compliance cultures. Consequently, the Turkish adaptation appears to reflect not a reduction of the original construct, but rather a culturally contextualized reorganization of motivational dimensions associated with safe care practices.

The psychometric findings obtained in this study further support the adequacy of the Turkish adaptation. The KMO coefficient demonstrated excellent sampling adequacy, while Bartlett's test of sphericity confirmed the suitability of the correlation matrix for factor analysis. These findings indicate that the data satisfied the statistical assumptions necessary to identify a stable multidimensional structure. Furthermore, the total explained variance of 62.4% can be considered satisfactory for a multidimensional psychometric instrument assessing complex educational, behavioural and attitudinal constructs. The resulting factorial structure demonstrated strong internal consistency, with Cronbach's *α* coefficients ranging between 0.798 and 0.942 across dimensions. These reliability levels indicate that the Turkish version of the instrument provides stable and coherent measurement across multiple conceptual domains associated with infection prevention and control.

The confirmatory factor analysis findings also supported the structural adequacy of the 13‐factor model obtained through exploratory analyses. The fit indices demonstrated an acceptable overall fit between the empirical data and the proposed multidimensional structure. Although covariance modifications were applied between several error terms during the modelling process, these modifications were theoretically justifiable and did not alter the conceptual integrity of the instrument. Overall, the CFA findings support the consistency between the empirical factorial solution and the theoretical dimensions underlying the InovSafeCare construct. These results reinforce the structural validity of the Turkish adaptation and support its applicability within nursing education settings.

Convergent and discriminant validity analyses additionally provided relevant evidence regarding the psychometric adequacy of the Turkish version of the scale. Composite reliability (CR) values remained above recommended thresholds across all dimensions, supporting satisfactory internal consistency throughout the multidimensional structure. Although several AVE values remained below the conventional 0.50 threshold, the corresponding CR coefficients exceeded acceptable levels. According to the recommendations of Fornell and Larcker (1981), convergent validity may still be considered acceptable under these conditions when construct reliability remains sufficiently high. Therefore, convergent validity was considered partially but adequately supported in the affected dimensions. In parallel, discriminant validity indicators demonstrated that most dimensions remained empirically distinguishable from one another, reinforcing the multidimensional and theoretically differentiated nature of the construct.

The test–retest analyses also demonstrated satisfactory temporal stability of the instrument. ICC findings indicated that the scale provides stable measurements over time both globally and across most subdimensions. Particularly high temporal consistency was observed in dimensions related to professional motivation and distracting clinical factors, suggesting that these constructs may represent relatively stable experiential and attitudinal domains among nursing students. Overall, the reliability findings indicate that the Turkish adaptation of the InovSafeCare Student Perception Scale provides stable, coherent and reproducible measurements across repeated administrations.

Taken together, the findings of this study support the validity and reliability of the Turkish version of the InovSafeCare Student Perception Scale and reinforce the applicability of the InovSafeCare framework across different cultural and educational settings. Beyond its psychometric contribution, the instrument may also provide significant practical utility for nursing education and infection prevention programmes. By identifying educational, motivational, behavioural and organisational dimensions associated with HCAI prevention and control, the scale may contribute to the development of safer learning environments, the improvement of clinical training strategies and the strengthening of patient safety cultures within undergraduate nursing education.

Finally, the present findings highlight the importance of continuing cross‐cultural evaluations of the InovSafeCare framework in different healthcare and educational systems. Future studies should continue exploring how sociocultural, institutional and professional socialization processes shape nursing students' infection prevention attitudes, motivations and compliance‐related behaviours. Longitudinal and cross‐national comparative studies may further contribute to understanding the stability, adaptability and predictive utility of the instrument across diverse contexts and healthcare training environments. This scale can be used in nursing education to assess students' infection control competencies, identify training needs, improve clinical practices and develop a patient safety culture.

### 4.1 Limitations

Several limitations should be considered when interpreting the findings of this study. First, although the sample size was statistically adequate for psychometric analyses, participants were recruited exclusively from nursing departments belonging to two health sciences faculties of the same university system. Consequently, the findings may not fully represent the diversity of nursing education environments, institutional cultures and clinical training contexts present across different universities and healthcare education systems in Türkiye.

Educational curricula, pedagogical approaches, clinical placement opportunities, institutional safety cultures, and professional socialization processes may vary substantially across universities and geographical regions. These contextual differences may influence nursing students' perceptions, motivations, attitudes, and infection prevention behaviours, potentially affecting the factorial organization and psychometric performance of the instrument in different populations. Therefore, future studies should continue evaluating the Turkish version of the InovSafeCare Student Perception Scale in larger and more heterogeneous samples drawn from different universities, healthcare institutions and sociocultural contexts.

In addition, the cross‐sectional design of the present study limits the possibility of examining longitudinal changes in nursing students' perceptions and infection prevention behaviours over time. Future longitudinal studies may contribute to assessing the temporal stability, predictive validity and educational sensitivity of the instrument throughout different stages of professional training and clinical exposure.

Despite these limitations, the present study provides important psychometric evidence supporting the validity, reliability, and cross‐cultural applicability of the Turkish version of the InovSafeCare Student Perception Scale.

## 5 Conclusion

This study aimed to adapt the InovSafeCare Scale into Turkish and evaluate its psychometric properties among undergraduate nursing students in the context of healthcare‐associated infection prevention and control.

The findings support the validity, reliability, and structural adequacy of the Turkish version of the InovSafeCare Student Perception Scale and confirm the applicability of the multidimensional InovSafeCare framework within the Turkish educational and clinical context. The resulting 13‐factor structure demonstrated satisfactory psychometric performance, including acceptable construct validity, internal consistency, convergent and discriminant validity and temporal stability.

Importantly, the present findings also suggest that the factorial organization of infection prevention‐related perceptions, motivations, and professional attitudes may be partially shaped by sociocultural, educational and institutional contexts. The partial reorganization of some dimensions observed during the adaptation process should therefore be interpreted not as a weakness of the instrument, but rather as evidence of the contextual sensitivity of the construct across different educational and healthcare systems.

Beyond its psychometric contribution, the Turkish adaptation of the InovSafeCare Student Perception Scale may also provide substantial practical utility for nursing education and patient safety initiatives. The instrument may support the identification of educational needs, motivational processes, behavioural risk factors, and institutional dimensions associated with infection prevention and control practices among nursing students. Consequently, the scale may contribute to the development of safer educational environments, the strengthening of infection prevention curricula and the promotion of patient safety cultures within undergraduate nursing education.

Finally, the present study reinforces the importance of continuing cross‐cultural evaluations of the InovSafeCare framework in different countries and healthcare training systems. Comparative international research may contribute not only to the further psychometric refinement of the instrument, but also to a broader understanding of how educational, cultural, and professional contexts influence nursing students' infection prevention attitudes, motivations, and safe care practices.

This scale can be used as a standard measurement tool for future interventional studies, evaluation of training programmes and research on infection control.

## Author Contributions

**Fatma Birgili:** conceptualization, methodology, data collection, data analysis, writing – original draft, writing – review and editing, supervision. **Nezihe Bulut Uğurlu:** writing – review and editing. **Amaia Yurrebaso:** conceptualization, methodology, data collection, writing – review and editing. **Güllü Yazkan:** data collection, writing – review and editing. All authors reviewed and approved the final manuscript.

## Funding

The authors have nothing to report.

## Ethics Statement

Permission was obtained from Muğla Sıtkı Koçman University Medical and Health Sciences Ethics Committee to conduct the research (Date: 30.09.2024, Protocol No: 240072, Decision No: 105 Number: 72867572‐050.01.04‐200). The patients/participants provided their written informed consent to participate in this study.

## Consent

Each participant was required to sign an informed consent form before being enrolled in the study and prior to any measurements being taken.

## Conflicts of Interest

The authors declare no conflicts of interest.

## Data Availability

The data that support the findings of this study are available from the corresponding author upon reasonable request.
